# Matrine and Oxymatrine: evaluating the gene mutation potential using *in silico* tools and the bacterial reverse mutation assay (Ames test)

**DOI:** 10.1093/mutage/gead032

**Published:** 2023-10-25

**Authors:** Benjamin Christian Fischer, Yemurai Musengi, Jeannette König, Benjamin Sachse, Stefanie Hessel-Pras, Bernd Schäfer, Carsten Kneuer, Kristin Herrmann

**Affiliations:** German Federal Institute for Risk Assessment, Department Pesticides Safety, 10589 Berlin, Berlin, Germany; German Federal Institute for Risk Assessment, Department Pesticides Safety, 10589 Berlin, Berlin, Germany; German Federal Institute for Risk Assessment, Department Pesticides Safety, 10589 Berlin, Berlin, Germany; German Federal Institute for Risk Assessment, Department Food Safety, 10589 Berlin, Berlin, Germany; German Federal Institute for Risk Assessment, Department Food Safety, 10589 Berlin, Berlin, Germany; German Federal Institute for Risk Assessment, Department Food Safety, 10589 Berlin, Berlin, Germany; German Federal Institute for Risk Assessment, Department Pesticides Safety, 10589 Berlin, Berlin, Germany; German Federal Institute for Risk Assessment, Department Pesticides Safety, 10589 Berlin, Berlin, Germany

**Keywords:** matrine, oxymatrine, mutagenicity, liquorice, Ames test, QSAR, *in silico*

## Abstract

The quinolizidine alkaloids matrine and its *N*-oxide oxymatrine occur in plants of the genus *Sophora*. Recently, matrine was sporadically detected in liquorice products. Morphological similarity of the liquorice plant *Glycyrrhiza glabra* with *Sophora* species and resulting confusion during harvesting may explain this contamination, but use of matrine as pesticide has also been reported. The detection of matrine in liquorice products raised concern as some studies suggested a genotoxic activity of matrine and oxymatrine. However, these studies are fraught with uncertainties, putting the reliability and robustness into question. Another issue was that *Sophora* root extracts were usually tested instead of pure matrine and oxymatrine. The aim of this work was therefore to determine whether matrine and oxymatrine have potential for causing gene mutations. In a first step and to support a weight-of-evidence analysis, *in silico* predictions were performed to improve the database using expert and statistical systems by VEGA, Leadscope (Instem^®^), and Nexus (Lhasa Limited). Unfortunately, the confidence levels of the predictions were insufficient to either identify or exclude a mutagenic potential. Thus, in order to obtain reliable results, the bacterial reverse mutation assay (Ames test) was carried out in accordance with OECD Test Guideline 471. The test set included the plate incorporation and the preincubation assay. It was performed with five different bacterial strains in the presence or absence of metabolic activation. Neither matrine nor oxymatrine induced a significant increase in the number of revertants under any of the selected experimental conditions. Overall, it can be concluded that matrine and oxymatrine are unlikely to have a gene mutation potential. Any positive findings with *Sophora* extracts in the Ames test may be related to other components. Notably, the results also indicated a need to extend the application domain of respective (Q)SAR tools to secondary plant metabolites.

## Introduction

**Figure 1.**
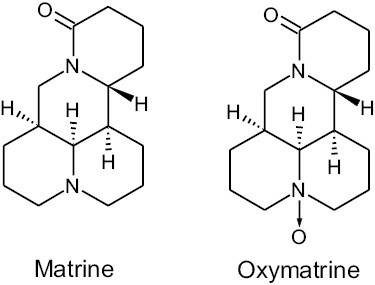
Chemical structures of matrine and its N-oxide oxymatrine.

The quinolizidine alkaloids matrine and its *N*-oxide oxymatrine occur naturally as main alkaloids in various plants of the genus *Sophora*, such as *Sophora flavescens* and *Sophora tonkinensis* [1,2]. Amounts of up to 1120 and 8770 mg/kg of matrine and oxymatrine, respectively, have been reported in samples of *S. flavescens* [3]. The chemical structures of both compounds are presented in Figure 1.

Dried roots of *Sophora* species are commonly used in Chinese traditional medicine for the treatment of several disorders, such as gastro-intestinal complaints and skin diseases [4]. In addition, several other beneficial effects, e.g. anti-inflammatory and anti-cancer effects, have also been attributed to *Sophora* roots, matrine, and oxymatrine [5–9]. In contrast, toxic effects, especially hepatotoxicity, neurotoxicity, and reproductive toxicity, have also been associated with exposure to these compounds [7,10,11].

Beside its utilization as a traditional medicine, matrine is used as a pesticide in Asian countries [12]. In the European Union, however, matrine and oxymatrine are considered as not approved pesticide active substances. No safety assessment has been conducted. For food control, a default maximum residue level (MRL) of 0.01 mg/kg applies [13,14].

Recently, the occurrence of matrine in concentrations above the default MRL was sporadically observed in liquorice products. As outlined by Schultz et al. [12], it is very unlikely that these findings are a result of matrine-containing pesticides, as the liquorice plant *Glycyrrhiza glabra* is not cultivated but wild growing. Since both plants are morphologically very similar, it rather appears plausible that liquorice plants are confused with *Sophora* species during harvesting. Consequently, matrine and oxymatrine in liquorice products may be considered as food contaminants. The same authors reported concentrations of up to 0.087 mg/kg matrine in powdered liquorice raw material [12].

As indicated in the publication by Schultz et al. the concentrations in liquorice products are relatively low [12]. However, low concentrations do not rule out the possibility of detrimental effects on human health from either substance. It should be mentioned that the toxicity profile of matrine and oxymatrine has not yet been characterized in detail. In particular, there is only limited data available addressing the genotoxic potential of these compounds.

Findings from some of the known studies suggest that there may be a concern for genotoxicity. However, most of the studies addressing this toxicological endpoint were conducted using *Sophora* extracts, as extracts are commonly used in Chinese traditional medicine. For example, Xue-jun et al. [15] investigated the genotoxicity of several herbal drugs used in Chinese traditional medicine using the bacterial reverse mutation test (Ames test). In the Ames test, conducted only with the two *Salmonella typhimurium* strains TA98 and TA100, positive results were obtained with a hot water extract of *Sophora japonica* in TA98 but not in TA100. In the same study the Ames test with hot water extracts of *S. flavescens* revealed no mutagenic findings [15]. Likewise, no mutagenic activity was observed with a hot water extract of *S. flavescens* in a study conducted by Che et al. in an Ames test performed according to OECD (Organisation for Economic Co-operation and Development) test guideline (TG) 471 [16]. Conflicting results were also obtained for other genotoxicity endpoints, such as clastogenicity [15–17]. A statistically significant increase of chromosomal aberrations and micronuclei was observed in the bone marrow of mice following intraperitoneal exposure to hot water extract of *Sophora* species [15]. In agreement, Che et al. [16] also found an increase of chromosomal aberrations in Chinese hamster lung fibroblasts in an *in vitro* test conducted according to OECD TG 473 with hot water extract of *S. flavescens* roots in the presence of a hepatic metabolic activation system. In contrast, no increased micronuclei formation was observed for the *S. flavescens* extract in an *in vivo* study performed according to OECD TG 474 on mice after gavage application of an extract. No activity was observed in a study conducted by Heo et al. [17] using the *in vivo* comet assay according to OECD TG 489 with hot water extract of *S. flavescens* and with pure matrine. In addition, albeit inconclusive the available studies raise some concern that *Sophora* root extracts may have a genotoxic potential, possibly due to the presence of matrine and oxymatrine. However, as non-characterized and standardized mixtures, which are highly questionable test articles for assessing the mutagenic potential of a single ingredient, were used in these experiments, it is not possible to reliably conclude on the genotoxic potential of the individual compounds matrine and oxymatrine. On the one hand, it cannot be excluded that negative results in some studies are due to the dilution of the active substances in the extract. On the other hand, genotoxic effects observed in some studies may have been mediated by the presence of other components. Currently, no studies are available addressing the mutagenic potential of pure matrine or oxymatrine.

Overall, the available data were not suitable to reliably conclude on the genotoxic potential of matrine and oxymatrine. In a first step, *in silico*, i.e. computational prediction models, were considered to generate supporting information. *In silico* tools are increasingly used in toxicology to support decision making in a weight of evidence approach. For example, OECD TG 497 on a Defined Approach for Skin Sensitisation builds on integrated testing strategies combining *in silico*, *in chemico*, and *in vitro* data [18]. The combination of experimental and *in silico* data has also been proposed for the assessment of genetic toxicity and can improve the overall robustness of an evaluation [19]. Prediction models for *in vitro* gene mutation are regarded as particularly well developed. Indeed, a validation exercise against an external dataset of pesticides and pesticide metabolites revealed for most tools a predictivity comparable with the intrinsic experimental variability of the Ames data, indicating a satisfactory performance of the models predicting mutagenicity in bacteria [20]. The models are generally divided into expert systems and statistical systems (QSAR). The approach of expert systems is based on the association of structural alerts and toxicological activity defined by rules (SAR). In contrast, QSAR systems use a statistical correlation between structural descriptors and toxicological activity [21]. In general, predictions of mutagenicity in bacteria from an expert system should be considered in conjunction with predictions by a QSAR system to increase the reliability of the prediction, some guidelines such as ICH M7 even specifically require complementary predictions [19,22]. Some models, such as *Caesar* by VEGA are so-called hybrid or integrated tools, which combine statistical and rule-based models to reduce the number of false negative predictions [23]. Consensus models also combine the output of two or more models, but these are not necessarily of different type. In this study, we included all of the above mentioned types of *in silico* models to generate information potentially supporting in the weight-of-evidence assessment for matrine and oxymatrine.

A more robust assessment of the genotoxic potential can be achieved by conducting appropriate tests following respective OECD guidelines. The current study evaluated the gene mutagenicity of both individual compounds utilizing the Ames test. The study was conducted in compliance with OECD TG 471. This test guideline defines the experimental framework for performing the Ames test for regulatory purposes. The Ames test is based on bacterial test strains with a deficiency in synthesizing a specific amino acid required for cell proliferation. Reverse mutations may occur after exposure to mutagenic test substances and thus, the test strains regain their ability to synthesize the essential amino acid. The test guideline includes requirements for bacterial strains to be tested, conditions to simulate liver metabolism (S9 liver fractions), and appropriate validated positive controls for each strain. In addition, concentration ranges for test substances are specified according to cytotoxic properties.

## Material and Methods

### (Q)SAR analysis for gene mutation potential

Following a combined approach, three different *in silico* applications, comprising complementary models (rule-based, statistical, and hybrid) were used to predict potential mutagenicity of matrine and oxymatrine. This included commercial as well as freely available software. Of note, all applied *in silico* tools are routinely used in regulatory practice. An overview can be found in Table 1.

**Table 1.** Commercial and freely available expert systems (SAR) and statistical systems (QSAR) for the prediction of mutagenicity *in vitro*

Tool	Model	Type	License
VEGA 1.1.5-b36	Mutagenicity consensus 1.0.3	Combination SAR + QSAR	Freely available
VEGA 1.1.5-b36	Caesar 2.1.13	Combination SAR + QSAR	Freely available
VEGA 1.1.5-b36	SarPy/IRFN 1.0.7	Expert system (SAR)	Freely available
VEGA 1.1.5-b36	ISS 1.0.2	Expert system (SAR)	Freely available
VEGA 1.1.5-b36	KNN/Read-Across 1.0.0	Statistical system (QSAR)	Freely available
Leadscope Model Applier 3.1.0-40	Genetic Toxicity Bacterial Mutation Alerts v8	Expert system (SAR)	Commercial
Leadscope Model Applier 3.1.0-40	Bacterial Mutation v2	Statistical system (QSAR)	Commercial
Leadscope Model Applier 3.1.0-40	E Coli—Sal 102 A-T Mut v2	Statistical system (QSAR)	Commercial
Leadscope Model Applier 3.1.0-40	Salmonella Mut v4	Statistical system (QSAR)	Commercial
Nexus 2.5.2	Derek 6.2.1	Expert system (SAR)	Commercial
Nexus 2.5.2	Sarah 3.2.1	Statistical system (QSAR)	Commercial

The publicly available tool VEGA 1.1.5-b36 was used to predict bacterial mutagenicity with the models *Caesar 2.1.13, SarPy/IRFN 1.0.7, ISS 1.0.2, KNN/Read-Across 1.0.0* as well as the *Mutagenicity consensus 1.0.3* model. Additionally, the Leadscope Model Applier 3.1.0-40 by Instem with the models *Genetic Toxicity Bacterial Mutation Alerts v8, Bacterial Mutation v2, E Coli—Sal 102 A-T Mut v2, Salmonella Mut v4* was applied as well as *Derek Nexus 6.2.1* and *Sarah Nexus 3.2.1* by Lhasa Limited. In total, a number of ten expert and statistical systems plus the VEGA consensus model were employed.

#### VEGA:

VEGA comprises four different models (*CEASAR*, *SarPy*, *ISS*, and *KNN/Read Across*) for the prediction of bacterial mutagenicity as well as a consensus model that provides an overall assessment based on the results and reliabilities of the individual models. KNN/Read Across and Caesar are statistical systems, the latter includes an expert system to reduce false-negative predictions. SarPy and ISS are expert systems. The four models provide information regarding the reliability of predictions (low, moderate, and high), whereas the consensus model expresses the reliability in form of a consensus score between 0 and 1, where ‘0’ means low reliability and ‘1’ high reliability. Detailed descriptions following the QSAR model reporting format (QMRF) are available at the respective webpage [24].

#### Leadscope:

The *Leadscope Model Applier* comprises four models (*Genetic Toxicity Bacterial Mutation Alerts, Bacterial Mutation, E Coli—Sal 102 A-T Mut, Salmonella Mut)* for the prediction of bacterial mutagenicity. The first model is an expert system, whereas the other three models are statistical systems. The model developers recommend to use the *Bacterial Mutation* model as the sole statistical system for the prediction of bacterial mutagenicity, being the more recent model based on an expanded dataset [25]. Accordingly, while all three statistical Leadscope models were applied, only the *Bacterial Mutation* model is considered in the analysis. The statistically based models of the software provide *Positive Probability* values ranging from 0 to 1, indicating a 0%–100% predicted likelihood of mutagenic potential based on a set of descriptors including but not limited to structural features of the query compound. In the expert model, the *Precision* is the number of true positives divided by the number of true positives plus the number of false positive and is used as a prediction parameter with values between 0.1352 and 1, with a value of 1 reflecting that 100% of substances in the reference dataset with the given alert are positive, whereas 0.1352 refers to the 13.52% of positive substances in the training data set that do not trigger any alerts. To conclude on the reliability of predictions by Leadscope, a number of additional elements are taken into account in an expert review, such as analysis of analogues and coverage of the structure of the query substance among others [26].

#### Derek and Sarah Nexus:

For the expert system Derek Nexus, genotoxicity in bacteria was selected as the toxicological endpoint. Sarah Nexus focuses per se only on the endpoint mutagenicity in bacteria.

Predictions using Derek Nexus with a likelihood level of at least equivocal were accepted. This implies that the hypothesis for or against mutagenic activity is supported or refuted by an equal number of arguments [27]. Regarding the Derek Nexus expert system, the data used to create alerts comes from a variety of sources. Among others this includes published literature, publicly available databases, proprietary data donations, knowledge transfer as well as data shared by consortia or members of Lhasa. The majority of data used for the derivation of structural alerts are from the pharmaceutical sector. However, the Derek knowledge base also includes information from other sources such as agrochemicals, foods or nutrition and cosmetics[28]. The training data set for the expert system Derek Nexus is not publicly available.

Among the variety of toxicological endpoints for which predictions by Derek nexus can be applied, one particular feature should be highlighted for the endpoint bacterial mutagenicity: It is nowadays possible to reliably perform negative predictions. Derek Nexus provides negative predictions in the absence of mutagenicity alerts to support expert assessment. The negative predictions functionality assesses all chemical features and compares them to a mutagenicity dataset. If there are no misclassified or unclassified features this is a highly confident negative prediction. Misclassified and unclassified features, however, require deeper expert review and the software makes this easy by highlighting the unknown or misclassified features within the dataset [29].

For the prediction with Sarah Nexus, the default reasoning type ‘weighted’ was selected as system setting. In addition, the parameters ‘equivocal’ and ‘sensitivity’ were, by default, each set to 8%. These settings have been agreed upon by scientists as well as regulators, as they promote a conservative approach to statistical mutagenicity assessment. Sarah Nexus identifies structural fragments from a training set that contains curated Ames data. Based on this, a hypothesis of *in vitro* mutagenicity is derived based on the structural fragment and the Ames test result of the parent compound [30]. Sarah Nexus predictions are based on non-confidential data and literature [31].

All *in silico* models used in this study fulfil the five OECD criteria for the validation of (Q)SAR models [32,33].

### Chemicals

The test substances matrine and oxymatrine were obtained from MedChemExpress, Monmouth Junction, New Jersey, USA. Purity as certified for batch no. 27619 (matrine) and 25568 (oxymatrine) was ≥ 98% for both substances. Certificates of analysis are provided as Supplementary Material. The positive controls 2-aminoanthracene, 2-nitrofluorene, 9-aminoacridine hydrochloride, benzo[*a*]pyrene, and cyclophosphamide mononitrate were obtained from Sigma-Aldrich, St. Louis, Missouri, USA. The solvent dimethyl sulfoxide (DMSO, dried) as well as disodium hydrogen phosphate anhydrous, magnesium chloride hexahydrate, potassium chloride, sodium azide, sodium chloride, sodium dihydrogen phosphate dihydrate, sodium ammonium hydrogen phosphate tetrahydrate, citric acid monohydrate, magnesium sulfate heptahydrate, di-potassium hydrogen phosphate, and l-tryptophane were obtained from Merck, Darmstadt, Germany. Ampicillin sodium salt, d(+)-glucose monohydrate, and nicotinamide adenine dinucleotide phosphate (NADP) disodium salt were obtained from AppliChem, Darmstadt, Germany. d-Glucose-6-phosphate disodium salt dihydrate and crystal violet indicator were obtained from Carl Roth, Karlsruhe, Germany. d(+)-Biotine and l-histidine hydrochloride monohydrate were obtained from neoLab, Heidelberg, Germany. Difco Nutrient Broth and Bacto Agar were obtained from BD Biosciences, Franklin Lakes, New Jersey, USA.

### Mutagenicity assay

To ensure a high quality, the bacterial reverse mutation test (Ames test) was conducted in accordance with OECD TG 471 [34] as described below.

### Colony counter

The bacterial colonies were counted using the Accu Count 1000 by Biosys Scientific Devices GmbH, Karben, Germany.

### Bacterial strains and metabolic activation

The tester strains *S. typhimurium* TA98, TA100, TA1535, and TA1537 as well as *E. coli* WP2 *uvrA* and rat liver S9 fraction (induced with phenobarbital/5,6-benzoflavone) were obtained from Moltox, Boone, North Carolina, USA. Each strain was checked by phenotypic testing for the presence of specific characteristics and mutations as recommended by OECD TG 471. In order to do so 100 µl of the bacterial culture were plated out in soft agar on complete agar plates. A filter paper soaked in either crystal violet (*rfa* mutation) or 0.5 mg/ml ampicillin (pKM101 plasmid) was plated into appropriately labelled plates before incubation upside down at 37°C for 48 h. For the *uvrB* mutation, the lid was removed; half of the plate was covered with aluminium foil and then exposed to UV light under the workbench at a distance of 30 cm for 1 min. After removal of the foil the plates were incubated upside down at 37 °C for 48 h.

### Preparation of test culture

An overnight culture was started by adding 0.2 ml of freshly thawed permanent culture to 20 ml liquid nutrient medium and incubated in a rotary incubator overnight for 7 h at 37°C while shaking at 200 rpm. The optical density of the overnight culture was determined after 7 h of incubation, before diluting it 1:20 with liquid nutrient medium (1 ml of overnight culture to 19 ml of liquid medium). Afterwards, incubation was continued for another 2.5 h in a rotary incubator at 37°C while shaking at 200 rpm. The optical density was determined immediately after dilution and again after 2 and 2.5 h to ensure a continuous bacterial growth. The bacterial solution was gradually diluted up to 1:10^6^ with phosphate buffer (0.1 M, pH 7.4) to determine the titre of the individual strains. Per complete agar plate 100 µl of the diluted cell suspension was plated (triplicate) and then incubated upside down at 37°C for 24–48 h. Thereby, a cell density of approximately 10^8^–10^9^ cells/ml was assured which is in line with the requirements of OECD TG 471.

### Plate incorporation test

The plate incorporation test was carried out using the *S. typhimurium* strains TA98, TA100, TA1535, and TA1537, together with *E. coli strain* WP2 *uvrA* with and without metabolic activation. DMSO was selected as solvent for matrine and oxymatrine. Preliminary tests were conducted in duplicate to determine the maximum test doses. No cytotoxicity or precipitation was observed with and without metabolic activation for doses up to 5000 µg/plate matrine or oxymatrine. Therefore, test doses of 312.5, 625, 1250, 2500, 3750, and 5000 µg/plate were used in the main experiment. Incubations were conducted in triplicate. For the experiment without metabolic activation 500 µl of phosphate buffer (0.1 M, pH 7.4) and for the experiment involving metabolic activation 500 µl of S9-mix (kept on ice) was added to labelled, sterile test tubes before adding 100 µl of bacterial suspension under sterile conditions. The S9-mix contained 10% S9-fraction (final concentration approx. 4 mg protein/ml), 100 mM Na_2_HPO_4_/NaH_2_PO_4_ buffer, 4 mM NADP (β-nicotinamide adenine dinucleotide phosphate disodium salt), 3 mM KCl, 8 mM MgCl_2_, and 5 mM glucose-6-phosphate. The test tubes were placed onto a 40°C thermoblock and then each test tube was taken off the block to add 50 µl of the respective matrine or oxymatrine dilution or phosphate buffer, DMSO (negative control) or appropriate positive control for the tested strain. Exactly 2 ml of top agar (6 mg/ml agar, 5 mg/ml NaCl, 50 µM biotin, 50 µM histidine/tryptophane) was added. Meanwhile the top agar maintained in a water bath at 42°C. Then the mixture was vortexed and plated onto minimal agar plates (15 mg/ml agar in Vogel-Bonner medium E with 22 mg/ml glucose). The plates were incubated upside down at 37°C for 48 h in the dark and afterwards the colonies (his^+^/tryp^+^ revertants) were counted using a colony counter. Additionally each plate was microscopically checked for potential background lawn changes indicating cytotoxicity.

### Pre-incubation test

A second test set was conducted to confirm the results of the plate-incorporation test following the pre-incubation test design. The same doses of matrine and oxymatrine were selected like in the plate incorporation test. Incubations were also conducted in triplicate. For the experiment without metabolic activation, 500 µl of phosphate buffer (0.1 M, pH 7.4) or for the experiment involving metabolic activation 500 µl of S9-mix (kept on ice) was added to all sterile test tubes of the triplicates before adding 100 µl of bacterial suspension. Then, 50 µl of the respective matrine or oxymatrine dilution or phosphate buffer, DMSO (negative control) or positive control was added at 30 s intervals. Afterwards, test tubes were placed onto a 40°C shaking thermoblock for 20 min each. The pipetting was done with increasing concentration and under and sterile conditions. After 20 min incubation, exactly 2 ml of top agar was added to the first test tube. Meanwhile, the top agar was maintained in a water bath at 42°C. Then, the mixture was vortexed, plated onto minimal agar plates, and allowed to set. This step was done at 30 s intervals to ensure incubation of each test tube for 20 min. The plates were incubated upside down at 37°C for 48 h. Afterward, the colonies were counted using a colony counter. Additionally each plate was checked by microscopy for potential changes of the background lawn indicating cytotoxicity.

## Results

### *In silico* predictions

The results of the predictions are summarized in Table 2. While metrics for the reliability of the predictions can vary a lot across different tools or individual models they are nonetheless provided in most cases. Comparing them directly may not be possible due to their various nature, but at least some semi-quantitative approach (low, moderate, and high) is appreciated to weigh different and/or contrary predictions. In addition, expert review is generally recommended, in particular in order to address conflicting calls [19].

**Table 2.** *In silico* predictions of different models regarding the endpoint bacterial mutagenicity for matrine and oxymatrine

		Matrine 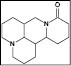		Oxymatrine 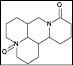	
Tool	Model	Prediction	Reliability	Prediction	Reliability
VEGA 1.1.5-b36	Mutagenicity consensus 1.0.3	non-mutagenic	low consensus score: 0.3	non-mutagenic	low consensus score: 0.3
VEGA 1.1.5-b36	Caesar 2.1.13	mutagenic	low global AD index = 0^#^	non-mutagenic	moderate global AD index = 0.803^#^
VEGA 1.1.5-b36	SarPy/IRFN 1.0.7	non-mutagenic	moderate global AD index = 0.894^#^	non-mutagenic	moderate global AD index = 0.803^#^
VEGA 1.1.5-b36	ISS 1.0.2	non-mutagenic	moderate global AD index = 0.749^#^	non-mutagenic	low global AD index = 0.625^#^
VEGA 1.1.5-b36	KNN/Read-Across 1.0.0	mutagenic	moderate global AD index = 0.771^#^	non-mutagenic	moderate global AD index = 0.766^#^
Leadscope Model Applier 3.1.0-40	Genetic Toxicity Bacterial Mutation Alerts v8	non-mutagenic	moderate^1^ precision: 0.1352^+^	not in domain	low^2^ precision: 0.1352^+^
Leadscope Model Applier 3.1.0-40	Bacterial Mutation v2	non-mutagenic	moderate^1^ PPP: 0.0334^*^	not in domain	low^2^ PPP: 0.0469^*^
Nexus 2.5.2	Derek 6.2.1	non-mutagenic	high no mcl or ucl	non-mutagenic	high no mcl or ucl
Nexus 2.5.2	Sarah 3.2.1	non-mutagenic	low confidence value: 16%	equivocal	n.a. confidence value: -
**Summary**		**inconclusive**		**inconclusive**	

^+^A precision of 0.1352 indicates that no alerts were identified and is thus the lowest precision that can be achieved. The precision of 0.1352 corresponds to 13.5% of substances in the training dataset that were positive without any alert and reflects the false-negative rate.

^*^The positive prediction probability (PPP) indicates the likelihood of a substance to be positive. A substance is predicted to be negative or positive, if the positive prediction probability is < 0.4 or > 0.6, respectively (maximum negative probability cut-off: 0.4; minimum positive probability cut-off: 0.6). .

^#^Global AD (applicability domain) index/consensus score < 0.7: low reliability; global AD index ≥ 0.7 and  < 0.9: moderate reliability; global AD index ≥ 0.9 and ≤ 1: high reliability; .

^1^Reliability after expert review.

^2^As the substance is not in domain, reliability is considered low.

Predictions and respective reliabilities of the different models have been standardized to a common terminology. n.a.: not applicable. Mcl/ucl: misclassified or unclassified features: No misclassified or unclassified features were hit which denotes that all structural features of the query compound are defined in the dataset.

#### Matrine:

VEGA’s consensus model as well as six out of 10 individual models predicted matrine to be non-mutagenic, two models predicted it to be mutagenic and for another two models it was outside the applicability domain, i.e. the structural features are not adequately represented in the training data set.

Notably, the reliability of the VEGA consensus model call as non-mutagenic can be regarded as low, considering the consensus score for non-mutagenicity of 0.3. This value is partially based on the contradicting positive prediction by VEGA`s Caesar model which, however, suffers from a low reliability (out of domain) in itself.

Another positive prediction was obtained with the statistical model VEGA KNN/Read-Across and reliability was described as moderate by the system. The model performs a read-across analysis based on experimental data for the (k) most similar molecules within the training dataset. Similarity is calculated taking into account not only the similarity coefficient (Tanimoto distance) between fingerprints as most common binary representations of the chemical structures but also further constitutional descriptors like number (and type) of atoms and number (and type) of bonds as well as—with lower weight— to information on the presence/absence of certain heteroatoms, functional groups etc. [35]. The positive call for matrine was based on two experimental positives in a small set of only four compounds showing a similarity value above the threshold of 0.8. When inspecting the structure of the two mutagenic substances, it appears that their activity may be related to confounding structural alerts that are not present in matrine: an N-Nitroso group in 1-nitrosoazacyclotridecane and a carbonyl chloride group in 1-chlorocarbonyl-4-piperidinopiperidine. Both functional groups are well-established alerts, but their presence in the source compounds used for the read-across had little impact due to low weighing (0.15) of the functional groups descriptor block.

The two remaining VEGA models reported negative predictions with moderate reliabilities. Of note, it should be considered that a low or moderate reliability indicates that the query compound is not well represented in the training data set.

In addition, negative predictions were also obtained using the expert model *Genetic Toxicity Bacterial Mutation Alerts v8* as well as the statistical model *Bacterial Mutation v2* by Leadscope. The negative prediction of the expert model is based on the absence of structural alerts of potential concern for mutagenicity, whereas the negative prediction of the statistical model is based on the low positive prediction probability. Expert review on the Leadscope models revealed, that in both models only one similar structure is included in the training/reference data set. The similarity between this analogue structure and matrine is considered relatively low with a Tanimoto score of 0.41, meaning that, unlike its molecular substructures, the entire structure of matrine is represented only to a limited extent in the model training/reference set. However, as no substructures of concern are identified, the reliability might be regarded as moderate.

Using the outdated Leadscope models *E Coli—Sal 102 A-T Mut, Salmonella Mut,* matrine was reported to be not in domain (results not shown in the Table 2).

The reliability (confidence) of the negative prediction by Sarah Nexus is low. Derek Nexus, however, provided a negative prediction with higher than moderate reliability.

#### Oxymatrine:

Regarding the results for oxymatrine, negative predictions appear to be predominant. Compared to matrine, however, oxymatrine appears to be even less well represented in the data sets although six models predicted oxymatrine to be non-mutagenic. The four VEGA models as well as the VEGA consensus model predicted oxymatrine to be non-mutagenic. With the exception of the *ISS* model (low reliability) VEGA predictions were of moderate reliability.

The four Leadscope models provided out of domain predictions for mutagenicity for oxymatrine due to the absence of sufficiently similar compounds in the model training/reference sets. Inspection of the results using the *Genetic Toxicity Bacterial Mutation Alerts* and *Bacterial Mutation* model highlighted that no alert was identified by the expert rule-based model, the statistical predictions have low positive prediction probabilities and the structure of oxymatrine, though not entirely represented, is to some extent covered by the model features. Thus, no specific concerns were identified. Nevertheless, from a regulatory perspective the confidence in the prediction results was limited due to the absence of similar structures in the training/reference sets. Overall, the reliability is considered to be low.

Derek Nexus predicted oxymatrine to be non-mutagenic with high confidence. Interestingly, the prediction for oxymatrine using Sarah was equivocal, meaning that there are arguments for and against a mutagenic activity. In the specific case of oxymatrine, the hypothesis generated by Sarah was positive due to the presence of the NO-group. However, this hypothesis was overruled by the most similar substances in the training set, as the majority of these compounds do not show a mutagenic activity. As the confidence of the overruled prediction being negative was very low at only 10%, the overall conclusion by Sarah was finally set as ‘*equivocal*’ without providing a confidence value.

All in all, both matrine and oxymatrine have been predicted as non-mutagenic by the majority of the applied models. However, both compounds have been predicted as positive or equivocal by at least one model. One may be inclined to weigh positive against negative predictions under consideration of their respective reliabilities. Thus, the negative predictions would outweigh the positive ones. However, this approach is not recommended as a single prediction of mutagenic potential triggered by a structural alert could indicate a concern for mutagenicity. For this reason, predictions for matrine and oyxmatrine were evaluated individually using expert judgement. The predictions showed low reliabilities, possibly due to inadequate representation of the query compound in the underlying training data set or due to conflicting experimental data of similar compounds in the training data set. Therefore, no reliable conclusion could be drawn.

In order to adequately address these uncertainties, we conducted Ames tests with the pure substances matrine and oxymatrine. To ensure high reliability of the test results, the experimental design was in accordance with OECD TG 471.

### Ames test

The Ames test was conducted with matrine and oxymatrine according to OECD TG 471 using five bacterial strains and two different study designs.

Positive control as well as background revertant counts were within the range of historical controls as reported by Levy et al., Hamel et al., Pant et al., and Kato et al. [36–39] for all strains in the absence and presence of S9 for both incubation types.

Tabular data are provided as individual data in Supplementary Material (Tables 3-6). Graphs illustrating the results for the plate incorporation method for matrine and oxymatrine, with and without metabolic activation are presented in the following section.

### Plate incorporation method

**Figure 2.**
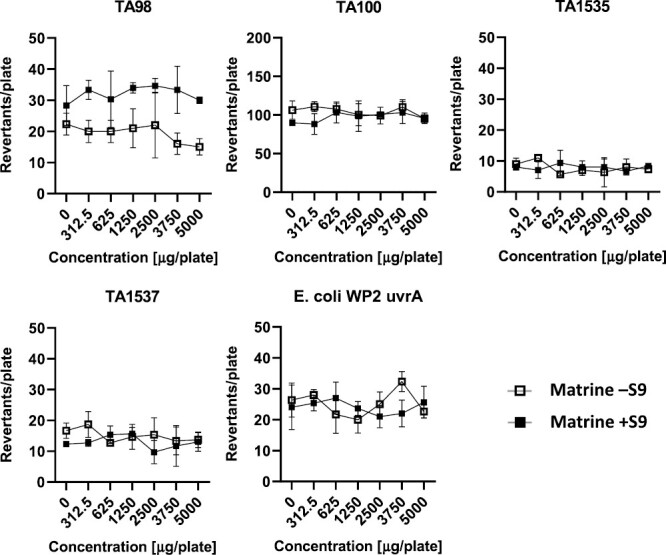
Results of the bacterial reverse mutation test of matrine for the five tester strains using the plate incorporation method, with and without metabolic activation. Revertant counts given as mean ± SD of three plates.

**Figure 3.**
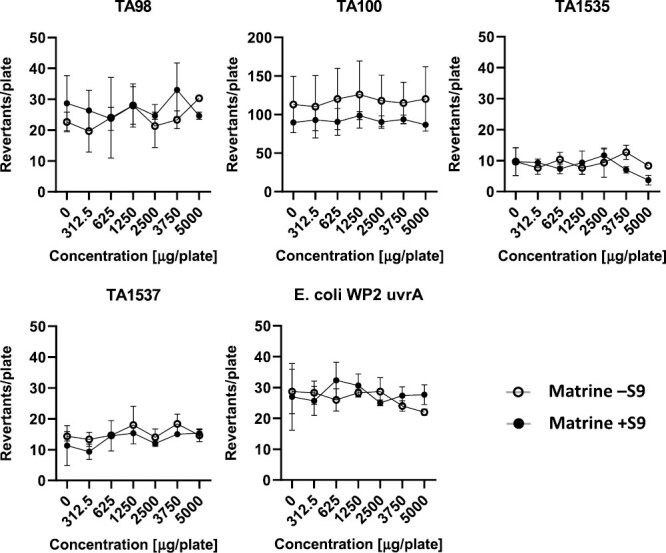
Results of the bacterial reverse mutation test of matrine for the five tester strains using the plate incorporation method, with and without metabolic activation. Revertant counts given as mean ± SD of three plates.

The quinolizidine alkaloids matrine and oxymatrine (purity ≥ 98%) were tested for their mutagenic potential. The mean revertant counts of the five tester strains treated with matrine and oxymatrine with and without S9-mix are given in Figure 2 and 3 respectively. No signs of cytotoxicity, indicated by a reduced background lawn or decreased revertant counts, or precipitation was observed for any of the tested strains up to the maximum test dose of 5000 µg/plate matrine or oxymatrine. A slight revertant count decrease compared to the solvent control was observed for matrine in TA98 without S9-mix, but was regarded as normal fluctuation, i.e. within the range of the solvent control reported in the literature [36]. No strain treated with matrine showed a dose-related or two-fold increase in revertant count, neither in the absence nor in the presence of metabolic activation. Matrine is therefore regarded as non-mutagenic in this test.

Similarly, the strains treated with oxymatrine did not show any signs of cytotoxicity or precipitation up to the dose of 5000 µg/plate with or without S9-mix. The decrease in revertants in TA1535 with S9-mix is not regarded as indicating cytotoxicity as the value is still within the range of the solvent control reported in the literature [36]. Furthermore, no dose-related or two-fold increase in revertants was observed, oxymatrine is therefore regarded to be non-mutagenic in this test.

### Pre-incubation method

In general, the results obtained with the pre-incubation method confirmed the findings from the plate incorporation test. Neither matrine nor oxymatrine induced a relevant increase in revertant colonies up to the highest tested dose of 5000 µg/plate. The responses of the positive control for the individual strains were in the expected ranges. Tabular data are shown in Supplementary Material.

## Discussion

Some studies with *Sophora* extracts have raised concern that matrine and oxymatrine may have genotoxic properties. Since the respective experimental studies were carried-out with non-characterized mixtures instead of pure substances and also had methodological deficiencies, the aim of this work was to investigate the mutagenic activity of matrine and oxymatrine. To this end, *in silico* studies as well as mutagenicity studies in bacteria were conducted.

### *In silico* predictions resulted in conflicting results

When using *in silico* tools, it is strongly recommended to combine two complementary systems with different algorithms (i.e. an expert with a statistical tool) to increase the sensitivity and reliability of the prediction [19,22,40,41]. Consumer health protection is the paramount objective in the field of pesticide regulation. In order to ensure a high level of safety it is preferable to maximise sensitivity, even at the expense of a decrease in specificity [22,42]. For this reason, different expert systems (SAR) and statistical (QSAR) systems were used in the current study to predict the bacterial mutagenicity of the plant-derived food contaminants matrine and oxymatrine.

As shown in Table 2, the *in silico* predictions are conflicting and showed different reliabilities, very likely due to the different training sets used and the methodologies.

Of all models applied, only Derek Nexus provided a prediction with a high reliability for matrine to be non-mutagenic. No misclassified or unclassified features were hit which denotes that all structural features of the query compound are defined in the dataset. Based on this prediction, Derek Nexus has found no cause for concern.

Non-mutagenicity of matrine was also predicted by the Leadscope models *Genetic Toxicity Bacterial Mutation Alerts v8* and *Bacterial Mutation v2*. A closer look at the prediction showed that despite the fact that matrine is limited represented in the model training/reference sets, the two complementary methodologies provided consistent negative outcomes and specific concerns were not identified. The expert review confirmed the negative prediction albeit with moderate reliability.

In line with matrine, only the prediction of oxymatrine with Derek Nexus showed a high reliability that it is non-mutagenic. Also, in this case no misclassified or unclassified features were matched.

Some of the *in silico* systems like Nexus provided high reliabilities to predict matrine and oxymatrine. This system covered the structural features and training data and can be considered as relevant for the prediction of mutagenicity. Due to different methodologies, the Leadscope models did not fully cover the chemical space to predict mutagenicity. For matrine, an expert review resulted into the classification non-mutagenic, demonstrating that the expert review is a key aspect of *in silico* evaluations. For oxymatrine, the Leadscope models were outside of the applicability domain, therefore further investigations, such as *in vitro* experiments and *in silico* predictions, are indicated.

Apart from the herein discussed case of matrine and oxymatrine, it has been shown that *in silico* predictions for the endpoint bacterial mutagenicity generally are sufficiently predictive. This was recently demonstrated for substance classes from the area of pesticide regulation [20,28]. Good model performance applies to both the prediction of mutagenic activity as well as the exclusion of potentially mutagenic properties at the bacterial level [29]. Thus, predictions by *in silico* systems for the endpoint mutagenicity in bacteria are widely accepted nowadays. However, the present case of matrine and oxymatrine exemplifies that an integrated approach using also experimental data may be necessary to achieve the required level of confidence. *In silico* data should not be evaluated in isolation, if the reliability of the predictions is low or if the results are conflicting or equivocal. Furthermore, even if time consuming, expert review of the predictions should be undertaken to increase the reliability of a conclusion. The case of matrine and oxymatrine highlights that collection and sharing of data remains essential to improve the training data sets of statistical models (QSAR) even for the endpoint of mutagenicity in bacteria. Matrine and oxymatrine are structurally very similar and differ only by an N-oxide group. The *in silico* analysis is able to take into account even small changes in the molecules. This may lead to out of domain or different predictions. It is well known in genotoxity testing, e.g. for nitroso compounds, that minor changes of the structure lead to different results in mutagenicity testing. This should be taken into account in any case when extending and expanding a training data set.

### Results of experimental data do not point to a mutagenic activity

The classical Ames test is used in many regulatory areas as an initial test to evaluate genotoxicity as it provides a quick and reliable indication of gene mutagenicity.

As shown in Figures 2 and 3, neither matrine nor oxymatrine led to a relevant increase in the number of revertants in the four *S. typhimurium* strains TA98, TA100, TA1535 and TA1537 as well as in the *E. coli* strain WP2 *uvrA*, neither with nor without metabolic activation.

Based on the experimental data presented in this study, it can be concluded that pure matrine and oxymatrine do not induce gene mutationsin bacteria. Thus, the positive findings observed in an Ames test with a hot water extract of *Sophora japonica* conducted by Xue-jun et al. [15] may be due to constituents other than matrine or oxymatrine.

### Other genotoxicity endpoints still need evaluation

However, besides gene mutagenicity, a number of studies conducted with *Sophora* extracts also point to a clastogenic potential, potentially attributed to matrine and oxymatrine as major constituents. For example, positive findings were observed in some studies for chromosomal aberrations and micronuclei [15,16]. Furthermore, one study is available that investigated the genotoxic potential of pure matrine using the *in vivo* comet assay according to OECD TG 489 [17]. The authors interpreted their findings as negative. However, it should be noted that the study suffers from some limitations and a slight dose-related increase in strand-breaks was actually observed. Thus, a further experimental evaluation of clastogenicity appears mandatory.

Noteworthy, *in silico* predictions for clastogenicity are currently not considered reliable. A possible explanation, among others, might be more complex adverse outcome pathways, different test protocols or diverging approaches for the evaluation and interpretation of test results [20,43,44]. Thus, *in silico* tools for the prediction of clastogenicity should rather be regarded as rough indication. Reliable predictions or even exclusion of clastogenicity is at the time being premature and experimental data are needed to draw reliable conclusions [45].

With respect to genotoxicity testing for risk assessment of substances in food and feed, the Scientific Committee of EFSA recommends the *in vitro* mammalian cell micronucleus test (OECD TG 487) as an initial step to evaluate clastogenic and/or aneugenic effects [41].

## Conclusions

The results of the *in silico* studies with both compounds showed that the reliability of predictions may be limited in cases where the substances under evaluation are not well covered by the training data. It is known that *in silico* models have limitations regarding their respective coverage of chemical space, but at least they usually report when compounds are not in the applicability domain. Assessment of the reliability based on the coverage of the individual structural features rather than the entire molecule may nevertheless be possible, as demonstrated for skin sensitization [46]. However, we did not consider this fragment-based approach as a generally accepted procedure in regulatory assessments. Experimental results are therefore required to expand the chemical space of the models and support predictions, if the query compounds are out of domain. Here we could demonstrate that neither matrine nor oxymatrine are mutagenic in the Ames test and experimental results essentially confirmed the output of Derek Nexus and supported the predictions of Sarah Nexus and Leadscope. Furthermore, the new Ames data can now be used to train and improve the systems.

Our study illustrates the need for integrated approaches combining *in silico* with experimental data, at least if the *in silico* predictions are considered insufficient.

## Supplementary Material

gead032_suppl_Supplementary_Material

## Data Availability

All data are incorporated into the article and its online supplementary material.

## References

[1] Li JC , Zhang ZJ, Liu D et al. Quinolizidine alkaloids from the roots of *Sophora flavescens*. Nat Prod Res 2022;36:1781–8. 32924588 10.1080/14786419.2020.1817011

[2] Ding, PI, Yu, YQ, Chen DF. Determination of quinolizidine alkaloids in Sophora tonkinensis by HPCE. Phytochem Anal 2005;16:257–63. 16042151 10.1002/pca.829

[3] Li K , Wang H. Simultaneous determination of matrine, sophoridine and oxymatrine in *Sophora flavescens* Ait by high performance liquid chromatography. Biomed Chromatogr 2004;18:178–82. 15103704 10.1002/bmc.308

[4] Schwarte, A. Phytochemische und pharmakologische Untersuchungen der Wurzeln von Sophora flavescens, unter besonderer Berücksichtigung ihrer Wirkung auf die Leukotrien- und Prostaglandinbiosynthese. Heinrich-Heine-Universität Düsseldorf: Institut für Pharmazeutische Biologie, 2002.

[5] Abd-Alla HI , Souguir D, Radwan MO. Genus Sophora: a comprehensive review on secondary chemical metabolites and their biological aspects from past achievements to future perspectives. Arch Pharm Res 2021;44:903–86. 34907492 10.1007/s12272-021-01354-2 PMC8671057

[6] Zhang H , Chen L, Sun X et al. Matrine: A promising natural product with various pharmacological activities. Front Pharmacol 2020;11:529–533. 32477114 10.3389/fphar.2020.00588 PMC7232545

[7] You L , Yang C, Du Y et al. A systematic review of the pharmacology, toxicology and pharmacokinetics of matrine. Front Pharmacol 2020;11:1–18. 33041782 10.3389/fphar.2020.01067 PMC7526649

[8] Li Y , Wang G, Liu J et al. Quinolizidine alkaloids derivatives from Sophora alopecuroides Linn: Bioactivities, structure-activity relationships and preliminary molecular mechanisms. Eur J Med Chem 2020;188:1–25. 10.1016/j.ejmech.2019.111972 31884408

[9] Lan X , Zhao J, Zhang Y et al. Oxymatrine exerts organ- and tissue-protective effects by regulating inflammation, oxidative stress, apoptosis, and fibrosis: From bench to bedside. Pharmacol Res 2020;151:1–12. 10.1016/j.phrs.2019.104541 31733326

[10] Gu LL , Shen ZL, Li YL et al. Oxymatrine causes hepatotoxicity by promoting the phosphorylation of JNK and induction of endoplasmic reticulum stress mediated by ROS in LO2 cells. Mol Cells 2018;41:401–12. 29754474 10.14348/molcells.2018.2180 PMC5974617

[11] Lu H , Zhang L, Gu L et al. Oxymatrine Induces Liver Injury through JNK Signalling Pathway Mediated by TNF-α In Vivo. Basic Clin Pharmacol Toxicol 2016;119:405–11. 27097917 10.1111/bcpt.12608

[12] Schultz J , Raters M, Wittig M et al. Analysis and occurrence of matrine in liquorice raw materials - Exclusion of its application as pesticide. Food Additives & Contaminants. Part A, Chemistry, Analysis, Control, Exposure & Risk Assessment 2022;39:351–61. 10.1080/19440049.2021.2005261 34883039

[13] European Commission. EU Pesticides Database - Oxymatrine. Brussels, Belgium: https://ec.europa.eu/food/plant/pesticides/eu-pesticides-database/start/screen/active-substances/details/1475, 2022.

[14] European Commission. EU Pesticides Database – Matrine. Brussels, Belgium: https://ec.europa.eu/food/plant/pesticides/eu-pesticides-database/start/screen/active-substances/details/1244, 2022.

[15] Xue-jun Y , De-xiang L, Hechuan W et al. A study on the mutagenicity of 102 raw pharmaceuticals used in Chinese traditional medicine . Mutat Res /Genet Toxicol 1991;260:73–82. 2027343

[16] Che J-H , Yun J-W, Kim Y-S et al. Genotoxicity and subchronic toxicity of Sophorae radix in rats: Hepatotoxic and genotoxic potential. Regul Toxicol Pharm: RTP 2015;71:379–87. 10.1016/j.yrtph.2015.01.012 25640205

[17] Heo S , Lee J, Jeon H et al. In vivo genotoxicity assessment of matrine and the water extract of sophorae radix using a comet assay. *J Food Hyg Saf* 2021;36:118–23.

[18] OECD. Guideline No. 497: Defined Approaches on Skin Sensitisation. Paris, France: Organisation for Economic Co-operation and Development, 2021.

[19] Hasselgren C , Ahlberg E, Akahori Y et al. Genetic toxicology in silico protocol. Regul Toxicol Pharmacol 2019;107:1–21. 10.1016/j.yrtph.2019.104403 PMC7485926 31195068

[20] Benigni R , Laura Battistelli C, Bossa C et al. Evaluation of the Applicability of Existing (q)sar Models for Predicting the Genotoxicity of Pesticides and Similarity Analysis Related with Genotoxicity of Pesticides for Facilitating of Grouping and Read Across.. Parma, Italy: EFSA Supporting Publications. 10.1016/j.yrtph.2020.104658 32334037

[21] OECD. Guidance onGrouping of Chemicals, 2nd. Paris, France: Organisation for Economic Co-operation and Development, 2017.

[22] EMA (2015) ICH Guideline M7 (2015) (R1) on Assessment and Control of DNA Reactive (Mutagenic) Impurities in Pharmaceuticals to Limit Potential Carcinogenic Risk. Amsterdam, Netherlands: European Medicines Agency.

[23] Ferrari T , Gini G. An open source multistep model to predict mutagenicity from statistical analysis and relevant structural alerts. Chem Cent J 2010;4:S2. 20678181 10.1186/1752-153X-4-S1-S2 PMC2913329

[24] (2023) https://www.vegahub.eu/portfolio-item/vega-qsar-models-qrmf/

[25] Landry C , Kim MT, Kruhlak NL et al. Transitioning to composite bacterial mutagenicity models in ICH M7 (Q)SAR analyses. Regul Toxicol Pharmacol 2019;109:104488. 31586682 10.1016/j.yrtph.2019.104488 PMC6919322

[26] Myatt GJ , Ahlberg E, Akahori Y et al. In silico toxicology protocols. Regul Toxicol Pharmacol 2018;96:1–17. 29678766 10.1016/j.yrtph.2018.04.014 PMC6026539

[27] Barber C , Hanser T, Judson P et al. Distinguishing between expert and statistical systems for application under ICH M7. Regul Toxicol Pharmacol 2017;84:124–30. 28057482 10.1016/j.yrtph.2016.12.012

[28] Herrmann K , Holzwarth A, Rime S et al. (Q)SAR tools for the prediction of mutagenic properties: Are they ready for application in pesticide regulation? Pest Manag Sci 2020;76:3316–25. 32223060 10.1002/ps.5828

[29] Williams RV , Amberg A, Brigo A et al. It's difficult, but important, to make negative predictions. Regul Toxicol Pharmacol 2016;76:79–86. 26785392 10.1016/j.yrtph.2016.01.008

[30] Hanser T , Barber C, Rosser E, Vessey JD, Webb SJ, and Werner S (2014) Self organising hypothesis networks: a new approach for representing and structuring SAR knowledge. *J Cheminformatics*, 6, 21. 10.1186/1758-2946-6-21 PMC4048587 24959206

[31] (2023) https://www.lhasalimited.org/solutions/in-silico-mutagenicity-assessment/

[32] (2021) https://www.lhasalimited.org/blog/how-derek-nexus-and-sarah-nexus-meet-the-5-oecd-principles/9971.

[33] OECD. OECD Principles for the Validation, for Regulatory Purpose, of (Q)SAR Models, 2014.

[34] OECD. Test No. 471: Bacterial Reverse Mutation Test. Paris, France: Organisation for Economic Co-operation and Development, 2020.

[35] Floris M , Manganaro A, Nicolotti O et al. A generalizable definition of chemical similarity for read-across. J Cheminf 2014;6:39. 10.1186/s13321-014-0039-1 PMC4212147 25383097

[36] Levy DD , Zeiger E, Escobar PA et al. Recommended criteria for the evaluation of bacterial mutagenicity data (Ames test). Mutat Res Genet Toxicol Environ Mutagen 2019;848:403074. 31708073 10.1016/j.mrgentox.2019.07.004

[37] Hamel A , Roy M, Proudlock R. The bacterial reverse mutation test. In: Proudlock R, ed., Genetic Toxicol Testing, Academic Press, Cambridge 2016:79–138.

[38] Pant K , Bruce S, Sly J et al. Bacterial mutagenicity assays: Vehicle and positive control results from the standard Ames assay, the 6- and 24-well miniaturized plate incorporation assays and the Ames II assay. Environ Mol Mutagen 2016;57:483–96. 27198925 10.1002/em.22014

[39] Kato M , Sugiyama KI, Fukushima T et al. Negative and positive control ranges in the bacterial reverse mutation test: JEMS/BMS collaborative study. Genes Environ 2018;40:7. 29632622 10.1186/s41021-018-0096-1 PMC5883876

[40] Worth A , Fuart-Gatnik M, Lapenna S et al. The use of computational methods in the toxicological assessment of chemicals in food: Current status and future prospects. EUR 24748 EN. Luxembourg (Luxembourg): Publications Office of the European Union; 2011. JRC63826.

[41] EFSA. Applicability of Qsar Analysis to the Evaluation of the Toxicological Relevance of Metabolites and Degradates of Pesticide Active Substances for Dietary Risk Assessment. Vol 7. EFSA Supporting Publications: 1–311, 2010.

[42] Wichard JD. In silico prediction of genotoxicity. Food Chem Toxicol 2017;106:595–9. 27979779 10.1016/j.fct.2016.12.013

[43] Benigni R , Serafimova R, Parra Morte JM et al. Evaluation of the applicability of existing (Q)SAR models for predicting the genotoxicity of pesticides and similarity analysis related with genotoxicity of pesticides for facilitating of grouping and read across: An EFSA funded project. Regul Toxicol Pharmacol 2020;114:104658. 32334037 10.1016/j.yrtph.2020.104658

[44] Benigni R , Bassan A, Pavan M. In silico models for genotoxicity and drug regulation. Expert Opin Drug Metab Toxicol 2020;16:651–62. 32567390 10.1080/17425255.2020.1785428

[45] Tcheremenskaia O , Benigni R. Toward regulatory acceptance and improving the prediction confidence of in silico approaches: a case study of genotoxicity. Expert Opin Drug Metab Toxicol 2021;17:987–1005. 34078212 10.1080/17425255.2021.1938540

[46] Chilton ML , Macmillan DS, Steger-Hartmann T, et al. Making reliable negative predictions of human skin sensitisation using an in silico fragmentation approach. Regul Toxicol Pharmacol 2018;95:227–35. 29580972 10.1016/j.yrtph.2018.03.015

