# Supplementary material for: Matrine and Oxymatrine: evaluating the gene mutation potential using in silico tools and the bacterial reverse mutation assay (Ames test)
Source: Mutagenesis. 2023 Oct 25;39(1):32–42. doi: 10.1093/mutage/gead032 (PMC10851102; doi:10.1093/mutage/gead032)
Supplement: gead032_suppl_Supplementary_Material [file gead032_suppl_supplementary_material.docx]

| **Table I.** Commercial and freely available Expert systems (SAR) and Statistical systems (QSAR) for the prediction of mutagenicity *in vitro* | | | |
| --- | --- | --- | --- |
| Tool | Model | Type | License |
| VEGA 1.1.5-b36 | Mutagenicity consensus 1.0.3 | Combination SAR+QSAR | freely available |
| VEGA 1.1.5-b36 | Caesar 2.1.13 | Combination SAR+QSAR | freely available |
| VEGA 1.1.5-b36 | SarPy/IRFN 1.0.7 | Expert system (SAR) | freely available |
| VEGA 1.1.5-b36 | ISS 1.0.2 | Expert system (SAR) | freely available |
| VEGA 1.1.5-b36 | KNN/Read-Across 1.0.0 | Statistical system (QSAR) | freely available |
| Leadscope Model Applier 3.1.0-40 | Genetic Toxicity Bacterial Mutation Alerts v8 | Expert system (SAR) | commercial |
| Leadscope Model Applier 3.1.0-40 | Bacterial Mutation v2 | Statistical system (QSAR) | commercial |
| Leadscope Model Applier 3.1.0-40 | E Coli - Sal 102 A-T Mut v2 | Statistical system (QSAR) | commercial |
| Leadscope Model Applier 3.1.0-40 | Salmonella Mut v4 | Statistical system (QSAR) | commercial |
| Nexus 2.5.2 | Derek 6.2.1 | Expert system (SAR) | commercial |
| Nexus 2.5.2 | Sarah 3.2.1 | Statistical system (QSAR) | commercial |

| **Table II.** *In silico* predictions of different models regarding the endpoint bacterial mutagenicity for matrine and oxymatrine. Predictions and respective reliabilities of the different models have been standardized to a common terminology | | | | | | | | |
| --- | --- | --- | --- | --- | --- | --- | --- | --- |
|  |  |  |  | Matrine   | |  | Oxymatrine | |
|  |  |  |  |  |  |  |  |  |
| Tool |  | Model |  | Prediction | Reliability |  | Prediction | Reliability |
| VEGA 1.1.5-b36 |  | Mutagenicity consensus 1.0.3 |  | non-mutagenic | low  consensus score: 0.3 |  | non-mutagenic | low  consensus score: 0.5 |
| VEGA 1.1.5-b36 |  | Caesar 2.1.13 |  | mutagenic | low  global AD index = 0^#^ |  | non-mutagenic | moderate  global AD index = 0.803^#^ |
| VEGA 1.1.5-b36 |  | SarPy/IRFN 1.0.7 |  | non-mutagenic | moderate  global AD index = 0.894^#^ |  | non-mutagenic | moderate  global AD index = 0.803^#^ |
| VEGA 1.1.5-b36 |  | ISS 1.0.2 |  | non-mutagenic | moderate  global AD index = 0.749^#^ |  | non-mutagenic | low  global AD index = 0.625^#^ |
| VEGA 1.1.5-b36 |  | KNN/Read-Across 1.0.0 |  | mutagenic | moderate  global AD index = 0.771^#^ |  | non-mutagenic | moderate  global AD index = 0.766^#^ |
| Leadscope Model Applier 3.1.0-40 |  | Genetic Toxicity Bacterial Mutation Alerts v8 |  | non-mutagenic | moderate^1^  precision: 0.1352^+^ |  | not in domain | low^2^  precision: 0.1352^+^ |
| Leadscope Model Applier 3.1.0-40 |  | Bacterial Mutation v2 |  | non-mutagenic | moderate^1^  PPP: 0.0334* |  | not in domain | low^2^  PPP: 0.0469* |
| Nexus 2.5.2 |  | Derek 6.2.1 |  | non-mutagenic  no mcl or ucl | high |  | non-mutagenic  no mcl or ucl | high |
| Nexus 2.5.2 |  | Sarah 3.2.1 |  | non-mutagenic | low  confidence value: 16% |  | equivocal | n.a.  confidence value: - |
| **Summary** | | |  | **inconclusive** |  |  | **inconclusive** |  |

^+^ A precision of 0.1352 indicates that no alerts were identified and is thus the lowest precision that can be achieved. The precision of 0.1352 corresponds to 13.5% of substances in the training dataset that were positive without any alert and reflects the false-negative rate.

* The positive prediction probability (PPP) indicates the likelihood of a substance to be positive. A substance is predicted to be negative or positive, if the positive prediction probability is < 0.4 or > 0.6, respectively (maximum negative probability cut-off: 0.4; minimum positive probability cut-off: 0.6).

^#^ global AD (applicability domain) index / consensus score < 0.7: low reliability; global AD index ≥0.7 and < 0.9: moderate reliability; global AD index ≥ 0.9 and ≤ 1: high reliability;

^1^ Reliability after expert review

^2^ As the substance is not in domain, reliability is considered low.

n.a.: not applicable

Mcl/ucl: misclassified or unclassified features: No misclassified or unclassified features were hit which denotes that all structural features of the query compound are defined in the dataset.

| **Table III.** Revertant counts in the plate incorporation experiment for matrine | | | | | | | | | | | | | | | |
| --- | --- | --- | --- | --- | --- | --- | --- | --- | --- | --- | --- | --- | --- | --- | --- |
|  |  | TA98 |  |  | TA100 |  |  | TA1535 | |  | TA1537 | |  | E.coli WP2 uvrA | |
|  |  | -S9 | +S9 |  | -S9 | +S9 |  | -S9 | +S9 |  | -S9 | +S9 |  | -S9 | +S9 |
| Matrine |  |  |  |  |  |  |  |  |  |  |  |  |  |  |  |
| 5000 µg/plate |  | 13 | 29 |  | 91 | 98 |  | 7 | 8 |  | 14 | 10 |  | 21 | 27 |
|  |  | 14 | 30 |  | 101 | 101 |  | 8 | 8 |  | 11 | 16 |  | 25 | 30 |
|  |  | 18 | 31 |  | 94 | 88 |  | 7 | 9 |  | 16 | 13 |  | 22 | 20 |
|  | Mean | 15 | 30 |  | 95.3 | 95.7 |  | 7.3 | 8.3 |  | 13.7 | 13.0 |  | 22.7 | 25.7 |
|  | SD | 2.6 | 1.0 |  | 5.1 | 6.8 |  | 0.6 | 0.6 |  | 2.5 | 3.0 |  | 2.1 | 5.1 |
|  | MF | 0.7 | 1.1 |  | 0.9 | 1.1 |  | 0.8 | 1.0 |  | 0.8 | 1.1 |  | 0.9 | 1.1 |
|  |  |  |  |  |  |  |  |  |  |  |  |  |  |  |  |
| Matrine |  |  |  |  |  |  |  |  |  |  |  |  |  |  |  |
| 3750 µg/plate |  | 12 | 42 |  | 106 | 114 |  | 9 | 8 |  | 9 | 6 |  | 30 | 27 |
|  |  | 18 | 28 |  | 104 | 108 |  | 10 | 6 |  | 18 | 19 |  | 36 | 19 |
|  |  | 18 | 30 |  | 121 | 87 |  | 5 | 6 |  | 13 | 10 |  | 31 | 20 |
|  | Mean | 16 | 33.3 |  | 110.3 | 103 |  | 8.0 | 6.7 |  | 13.3 | 11.7 |  | 32.3 | 22.0 |
|  | SD | 3.5 | 7.6 |  | 9.3 | 14.2 |  | 2.6 | 1.2 |  | 4.5 | 6.7 |  | 3.2 | 4.4 |
|  | MF | 0.7 | 1.2 |  | 1.0 | 1.1 |  | 0.9 | 0.8 |  | 0.8 | 0.9 |  | 1.2 | 0.9 |
|  |  |  |  |  |  |  |  |  |  |  |  |  |  |  |  |
| Matrine |  |  |  |  |  |  |  |  |  |  |  |  |  |  |  |
| 2500 µg/plate |  | 23 | 32 |  | 87 | 101 |  | 8 | 10 |  | 18 | 8 |  | 29 | 24 |
|  |  | 32 | 36 |  | 107 | 100 |  | 10 | 5 |  | 19 | 14 |  | 25 | 17 |
|  |  | 11 | 36 |  | 104 | 101 |  | 1 | 9 |  | 9 | 7 |  | 21 | 22 |
|  | Mean | 22 | 34.7 |  | 99.3 | 100.7 |  | 6.3 | 8.0 |  | 15.3 | 9.7 |  | 25.0 | 21.0 |
|  | SD | 10.5 | 2.3 |  | 10.8 | 0.6 |  | 4.7 | 2.6 |  | 5.5 | 3.8 |  | 4.0 | 3.6 |
|  | MF | 1.0 | 1.2 |  | 0.9 | 1.1 |  | 0.7 | 1.0 |  | 0.9 | 0.8 |  | 0.9 | 0.9 |
|  |  |  |  |  |  |  |  |  |  |  |  |  |  |  |  |
| Matrine |  |  |  |  |  |  |  |  |  |  |  |  |  |  |  |
| 1250 µg/plate |  | 28 | 35 |  | 103 | 121 |  | 8 | 6 |  | 19 | 15 |  | 18 | 25 |
|  |  | 19 | 32 |  | 85 | 89 |  | 8 | 10 |  | 11 | 14 |  | 17 | 25 |
|  |  | 16 | 35 |  | 113 | 85 |  | 5 | 8 |  | 14 | 18 |  | 25 | 21 |
|  | Mean | 21 | 34 |  | 100.3 | 98.3 |  | 7.0 | 8.0 |  | 14.7 | 15.7 |  | 20.0 | 23.7 |
|  | SD | 6.2 | 1.7 |  | 14.2 | 19.7 |  | 1.7 | 2.0 |  | 4.0 | 2.1 |  | 4.4 | 2.3 |
|  | MF | 0.9 | 1.2 |  | 0.9 | 1.1 |  | 0.8 | 1.0 |  | 0.9 | 1.3 |  | 0.8 | 1.0 |
|  |  |  |  |  |  |  |  |  |  |  |  |  |  |  |  |
| Matrine |  |  |  |  |  |  |  |  |  |  |  |  |  |  |  |
| 625 µg/plate |  | 23 | 34 |  | 99 | 105 |  | 5 | 6 |  | 13 | 17 |  | 27 | 24 |
|  |  | 16 | 20 |  | 112 | 89 |  | 6 | 14 |  | 13 | 12 |  | 15 | 24 |
|  |  | 21 | 37 |  | 112 | 116 |  | 6 | 8 |  | 12 | 17 |  | 23 | 33 |
|  | Mean | 20 | 30.3 |  | 107.7 | 103.3 |  | 5.7 | 9.3 |  | 12.7 | 15.3 |  | 21.7 | 27.0 |
|  | SD | 3.6 | 9.1 |  | 7.5 | 13.6 |  | 0.6 | 4.2 |  | 0.6 | 2.9 |  | 6.1 | 5.2 |
|  | MF | 0.9 | 1.1 |  | 1.0 | 1.2 |  | 0.6 | 1.2 |  | 0.8 | 1.2 |  | 0.8 | 1.1 |
|  |  |  |  |  |  |  |  |  |  |  |  |  |  |  |  |
| Matrine |  |  |  |  |  |  |  |  |  |  |  |  |  |  |  |
| 312.5 µg/plate |  | 17 | 34 |  | 107 | 88 |  | 12 | 4 |  | 22 | 12 |  | 29 | 28 |
|  |  | 19 | 30 |  | 107 | 75 |  | 10 | 9 |  | 20 | 12 |  | 26 | 25 |
|  |  | 24 | 36 |  | 118 | 102 |  | 11 | 8 |  | 14 | 14 |  | 29 | 23 |
|  | Mean | 20 | 33.3 |  | 110.7 | 88.3 |  | 11.0 | 7.0 |  | 18.7 | 12.7 |  | 28.0 | 25.3 |
|  | SD | 3.6 | 3.1 |  | 6.4 | 13.5 |  | 1.0 | 2.6 |  | 4.2 | 1.2 |  | 1.7 | 2.5 |
|  | MF | 0.9 | 1.2 |  | 1.0 | 1.0 |  | 1.2 | 0.9 |  | 1.1 | 1.0 |  | 1.1 | 1.1 |
|  |  |  |  |  |  |  |  |  |  |  |  |  |  |  |  |
| Solvent control |  |  |  |  |  |  |  |  |  |  |  |  |  |  |  |
|  |  | 22 | 33 |  | 101 | 87 |  | 7 | 9 |  | 14 | 12 |  | 20 | 16 |
|  |  | 26 | 21 |  | 120 | 92 |  | 9 | 7 |  | 17 | 13 |  | 30 | 26 |
|  |  | 19 | 31 |  | 98 | 90 |  | 11 | 8 |  | 19 | 12 |  | 29 | 30 |
|  | Mean | 22.3 | 28.3 |  | 106.3 | 89.7 |  | 9.0 | 8.0 |  | 16.7 | 12.3 |  | 26.3 | 24.0 |
|  | SD | 3.5 | 6.4 |  | 11.9 | 2.5 |  | 2.0 | 1.0 |  | 2.5 | 0.6 |  | 5.5 | 7.2 |
|  | MF | 1.0 | 1.0 |  | 1.0 | 1.0 |  | 1.0 | 1.0 |  | 1.0 | 1.0 |  | 1.0 | 1.0 |
|  |  |  |  |  |  |  |  |  |  |  |  |  |  |  |  |
| Positive control |  |  |  |  |  |  |  |  |  |  |  |  |  |  |  |
|  |  | 489 | 925 |  | 667 | 930 |  | 482 | 93 |  | 380 | 108 |  | 502 | 125 |
|  |  | 549 | 954 |  | 645 | 950 |  | 490 | 76 |  | 673 | 111 |  | 440 | 120 |
|  |  | 563 | 849 |  | 574 | 987 |  | 414 | 86 |  | 557 | 103 |  | 422 | 107 |
|  | Mean | 533.7 | 909.3 |  | 628.7 | 955.7 |  | 462.0 | 85.0 |  | 536.7 | 107.3 |  | 454.7 | 117.3 |
|  | SD | 39.3 | 54.2 |  | 48.6 | 28.9 |  | 41.8 | 8.5 |  | 147.6 | 4.0 |  | 42.0 | 9.3 |
|  | MF | 23.9 | 32.1 |  | 5.9 | 10.7 |  | 51.3 | 10.6 |  | 32.2 | 8.7 |  | 17.3 | 4.9 |
|  |  | 2-NF | 2-AA |  | NaN_3_ | 2-AA |  | NaN_3_ | 2-AA |  | 9-AA | 2-AA |  | 4-NQO | 2-AA |
|  | µg/plate | 4 | 2 |  | 2.5 | 2 |  | 2.5 | 2 |  | 50 | 2 |  | 1 | 10 |

| **Table IV.** Revertant counts in the plate incorporation experiment for oxymatrine | | | | | | | | | | | | | | | |
| --- | --- | --- | --- | --- | --- | --- | --- | --- | --- | --- | --- | --- | --- | --- | --- |
|  |  | TA98 |  |  | TA100 |  |  | TA1535 | |  | TA1537 | |  | E.coli WP2 uvrA | |
|  |  | -S9 | +S9 |  | -S9 | +S9 |  | -S9 | +S9 |  | -S9 | +S9 |  | -S9 | +S9 |
| Oxymatrine |  |  |  |  |  |  |  |  |  |  |  |  |  |  |  |
| 5000 µg/plate |  | 31 | 24 |  | 168 | 84 |  | 8 | 5 |  | 14 | 16 |  | 23 | 30 |
|  |  | 30 | 26 |  | 103 | 88 |  | 8 | 2 |  | 13 | 16 |  | 22 | 24 |
|  |  | 30 | 24 |  | 90 | 88 |  | 9 | 4 |  | 17 | 14 |  | 21 | 29 |
|  | Mean | 30.3 | 24.7 |  | 120.3 | 86.7 |  | 8.3 | 3.7 |  | 14.7 | 15.3 |  | 22.0 | 27.7 |
|  | SD | 0.6 | 1.2 |  | 41.8 | 2.3 |  | 0.6 | 1.5 |  | 2.1 | 1.2 |  | 1.0 | 3.2 |
|  | MF | 1.3 | 0.9 |  | 1.1 | 1.0 |  | 0.9 | 0.4 |  | 1.0 | 1.4 |  | 0.8 | 1.0 |
|  |  |  |  |  |  |  |  |  |  |  |  |  |  |  |  |
| Oxymatrine |  |  |  |  |  |  |  |  |  |  |  |  |  |  |  |
| 3750 µg/plate |  | 25 | 43 |  | 146 | 94 |  | 14 | 8 |  | 17 | 15 |  | 23 | 24 |
|  |  | 20 | 27 |  | 102 | 88 |  | 10 | 6 |  | 22 | 15 |  | 26 | 29 |
|  |  | 25 | 29 |  | 97 | 99 |  | 14 | 7 |  | 16 | 15 |  | 23 | 29 |
|  | Mean | 23.3 | 33.0 |  | 115.0 | 93.7 |  | 12.7 | 7.0 |  | 18.3 | 15.0 |  | 24.0 | 27.3 |
|  | SD | 2.9 | 8.7 |  | 27.0 | 5.5 |  | 2.3 | 1.0 |  | 3.2 | 0.0 |  | 1.7 | 2.9 |
|  | MF | 1.0 | 1.2 |  | 1.0 | 1.0 |  | 1.3 | 0.7 |  | 1.3 | 1.3 |  | 0.8 | 1.0 |
|  |  |  |  |  |  |  |  |  |  |  |  |  |  |  |  |
| Oxymatrine |  |  |  |  |  |  |  |  |  |  |  |  |  |  |  |
| 2500 µg/plate |  | 14 | 24 |  | 156 | 94 |  | 13 | 10 |  | 16 | 13 |  | 24 | 26 |
|  |  | 28 | 24 |  | 104 | 96 |  | 4 | 14 |  | 15 | 11 |  | 29 | 25 |
|  |  | 22 | 26 |  | 94 | 81 |  | 11 | 11 |  | 11 | 12 |  | 33 | 24 |
|  | Mean | 21.3 | 24.7 |  | 118 | 90.3 |  | 9.3 | 11.7 |  | 14.0 | 12.0 |  | 28.7 | 25.0 |
|  | SD | 7.0 | 1.2 |  | 33.3 | 8.1 |  | 4.7 | 2.1 |  | 2.6 | 1.0 |  | 4.5 | 1.0 |
|  | MF | 0.9 | 0.9 |  | 1.0 | 1.0 |  | 1.0 | 1.2 |  | 1.0 | 1.1 |  | 1.0 | 0.9 |
|  |  |  |  |  |  |  |  |  |  |  |  |  |  |  |  |
| Oxymatrine |  |  |  |  |  |  |  |  |  |  |  |  |  |  |  |
| 1250 µg/plate |  | 24 | 20 |  | 176 | 104 |  | 7 | 5 |  | 15 | 15 |  | 27 | 28 |
|  |  | 25 | 33 |  | 105 | 94 |  | 10 | 12 |  | 25 | 16 |  | 29 | 29 |
|  |  | 35 | 31 |  | 97 | 98 |  | 6 | 11 |  | 14 | 15 |  | 29 | 35 |
|  | Mean | 28 | 28 |  | 126 | 98.7 |  | 7.7 | 9.3 |  | 18.0 | 15.3 |  | 28.3 | 30.7 |
|  | SD | 6.1 | 7.0 |  | 43.5 | 5.0 |  | 2.1 | 3.8 |  | 6.1 | 0.6 |  | 1.2 | 3.8 |
|  | MF | 1.2 | 1.0 |  | 1.1 | 1.1 |  | 0.8 | 1.0 |  | 1.3 | 1.4 |  | 1.0 | 1.1 |
|  |  |  |  |  |  |  |  |  |  |  |  |  |  |  |  |
| Oxymatrine |  |  |  |  |  |  |  |  |  |  |  |  |  |  |  |
| 625 µg/plate |  | 22 | 21 |  | 166 | 89 |  | 13 | 6 |  | 15 | - |  | 23 | 39 |
|  |  | 38 | 22 |  | 102 | 74 |  | 9 | 9 |  | 15 | 11 |  | 25 | 28 |
|  |  | 12 | 28 |  | 93 | 109 |  | 9 | 7 |  | 14 | 18 |  | 30 | 30 |
|  | Mean | 24 | 23.7 |  | 120.3 | 90.7 |  | 10.3 | 7.3 |  | 14.7 | 14.5 |  | 26.0 | 32.3 |
|  | SD | 13.1 | 3.8 |  | 39.8 | 17.6 |  | 2.3 | 1.5 |  | 0.6 | 4.9 |  | 3.6 | 5.9 |
|  | MF | 1.1 | 0.8 |  | 1.1 | 1.0 |  | 1.1 | 0.8 |  | 1.0 | 1.3 |  | 0.9 | 1.2 |
|  |  |  |  |  |  |  |  |  |  |  |  |  |  |  |  |
| Oxymatrine |  |  |  |  |  |  |  |  |  |  |  |  |  |  |  |
| 312.5 µg/plate |  | 12 | 20 |  | 157 | 93 |  | 6 | 10 |  | 12 | 7 |  | 24 | 31 |
|  |  | 22 | 26 |  | 83 | 79 |  | 7 | 10 |  | 12 | 12 |  | 31 | 24 |
|  |  | 25 | 33 |  | 91 | 107 |  | 10 | 8 |  | 16 | 9 |  | 30 | 22 |
|  | Mean | 19.7 | 26.3 |  | 110.3 | 93.0 |  | 7.7 | 9.3 |  | 13.3 | 9.3 |  | 28.3 | 25.7 |
|  | SD | 6.8 | 6.5 |  | 40.6 | 14.0 |  | 2.1 | 1.2 |  | 2.3 | 2.5 |  | 3.8 | 4.7 |
|  | MF | 0.9 | 0.9 |  | 1.0 | 1.0 |  | 0.8 | 1.0 |  | 0.9 | 0.8 |  | 1.0 | 1.0 |
|  |  |  |  |  |  |  |  |  |  |  |  |  |  |  |  |
| Solvent control |  |  |  |  |  |  |  |  |  |  |  |  |  |  |  |
|  |  | 19 | 24 |  | 152 | 90 |  | 10 | 10 |  | 16 | 5 |  | 24 | 36 |
|  |  | 25 | 23 |  | 107 | 92 |  | 5 | 14 |  | 14 | 11 |  | 37 | 15 |
|  |  | 24 | 39 |  | 80 | 87 |  | 14 | 5 |  | 13 | 18 |  | 25 | 30 |
|  | Mean | 22.7 | 28.7 |  | 113.0 | 89.7 |  | 9.7 | 9.7 |  | 14.3 | 11.3 |  | 28.7 | 27.0 |
|  | SD | 3.2 | 9.0 |  | 36.4 | 2.5 |  | 4.5 | 4.5 |  | 1.5 | 6.5 |  | 7.2 | 10.8 |
|  | MF | 1.0 | 1.0 |  | 1.0 | 1.0 |  | 1.0 | 1.0 |  | 1.0 | 1.0 |  | 1.0 | 1.0 |
|  |  |  |  |  |  |  |  |  |  |  |  |  |  |  |  |
| Positive control |  |  |  |  |  |  |  |  |  |  |  |  |  |  |  |
|  |  | 486 | 991 |  | 219 | 884 |  | 604 | 88 |  | 673 | 98 |  | 450 | 112 |
|  |  | 491 | 837 |  | 348 | 790 |  | 571 | 89 |  | 553 | 90 |  | 476 | 125 |
|  |  | 358 | 702 |  | 172 | 755 |  | 569 | 70 |  | 497 | 122 |  | 477 | 129 |
|  | Mean | 445.0 | 843.3 |  | 246.3 | 809.7 |  | 581.3 | 82.3 |  | 574.3 | 103.3 |  | 467.7 | 122.0 |
|  | SD | 75.4 | 144.6 |  | 91.1 | 66.7 |  | 19.7 | 10.7 |  | 89.9 | 16.7 |  | 15.3 | 8.9 |
|  | MF | 19.6 | 29.4 |  | 2.2 | 9.0 |  | 60.1 | 8.5 |  | 40.1 | 9.1 |  | 16.3 | 4.5 |
|  |  | 2-NF | 2-AA |  | NaN_3_ | 2-AA |  | NaN_3_ | 2-AA |  | 9-AA | 2-AA |  | 4-NQO | 2-AA |
|  | µg/plate | 4 | 2 |  | 2.5 | 2 |  | 2.5 | 2 |  | 50 | 2 |  | 1 | 10 |

| **Table V.** Revertant counts in the pre-incubation experiment for matrine | | | | | | | | | | | | | | | |
| --- | --- | --- | --- | --- | --- | --- | --- | --- | --- | --- | --- | --- | --- | --- | --- |
|  |  | TA98 |  |  | TA100 |  |  | TA1535 | |  | TA1537 | |  | E.coli WP2 uvrA | |
|  |  | -S9 | +S9 |  | -S9 | +S9 |  | -S9 | +S9 |  | -S9 | +S9 |  | -S9 | +S9 |
| Matrine |  |  |  |  |  |  |  |  |  |  |  |  |  |  |  |
| 5000 µg/plate |  | 12 | 29 |  | 53 | 75 |  | 12 | 18 |  | 19 | 16 |  | 16 | 14 |
|  |  | 13 | 34 |  | 47 | 83 |  | 14 | 13 |  | 25 | 16 |  | 18 | 28 |
|  |  | 12 | 24 |  | 86 | 75 |  | 8 | 13 |  | 16 | 10 |  | 29 | 22 |
|  | Mean | 12.3 | 29 |  | 62.0 | 77.7 |  | 11.3 | 14.7 |  | 20.0 | 14.0 |  | 21.0 | 21.3 |
|  | SD | 0.6 | 5.0 |  | 21.0 | 4.6 |  | 3.1 | 2.9 |  | 4.6 | 3.5 |  | 7.0 | 7.0 |
|  | MF | 0.9 | 0.9 |  | 0.6 | 0.8 |  | 0.6 | 1.0 |  | 1.0 | 1.0 |  | 0.7 | 0.8 |
|  |  |  |  |  |  |  |  |  |  |  |  |  |  |  |  |
| Matrine |  |  |  |  |  |  |  |  |  |  |  |  |  |  |  |
| 3750 µg/plate |  | 12 | 33 |  | 125 | 69 |  | 12 | 15 |  | 21 | 11 |  | 26 | 29 |
|  |  | 14 | 22 |  | 98 | 90 |  | 15 | 15 |  | 19 | 14 |  | 19 | 28 |
|  |  | 10 | 32 |  | 121 | 96 |  | 18 | 14 |  | 28 | 17 |  | 22 | 23 |
|  | Mean | 12 | 29.0 |  | 114.7 | 85 |  | 15.0 | 14.7 |  | 22.7 | 14.0 |  | 22.3 | 26.7 |
|  | SD | 2.0 | 6.1 |  | 14.6 | 14.2 |  | 3.0 | 0.6 |  | 4.7 | 3.0 |  | 3.5 | 3.2 |
|  | MF | 0.8 | 0.9 |  | 1.2 | 0.9 |  | 0.8 | 1.0 |  | 1.1 | 1.0 |  | 0.7 | 1.0 |
|  |  |  |  |  |  |  |  |  |  |  |  |  |  |  |  |
| Matrine |  |  |  |  |  |  |  |  |  |  |  |  |  |  |  |
| 2500 µg/plate |  | 12 | 24 |  | 101 | 99 |  | 16 | 11 |  | 17 | 14 |  | 28 | 32 |
|  |  | 9 | 24 |  | 98 | 100 |  | 14 | 10 |  | 16 | 11 |  | 26 | 22 |
|  |  | 9 | 24 |  | 93 | 107 |  | 16 | 12 |  | 23 | 19 |  | 26 | 31 |
|  | Mean | 10 | 24.0 |  | 97.3 | 102.0 |  | 15.3 | 11.0 |  | 18.7 | 14.7 |  | 26.7 | 28.3 |
|  | SD | 1.7 | 0.0 |  | 4.0 | 4.4 |  | 1.2 | 1.0 |  | 3.8 | 4.0 |  | 1.2 | 5.5 |
|  | MF | 0.7 | 0.7 |  | 1.0 | 1.1 |  | 0.8 | 0.8 |  | 0.9 | 1.0 |  | 0.9 | 1.0 |
|  |  |  |  |  |  |  |  |  |  |  |  |  |  |  |  |
| Matrine |  |  |  |  |  |  |  |  |  |  |  |  |  |  |  |
| 1250 µg/plate |  | 10 | 25 |  | 123 | 87 |  | 12 | 14 |  | 22 | 8 |  | 34 | 25 |
|  |  | 14 | 24 |  | 105 | 80 |  | 17 | 14 |  | 20 | 12 |  | 22 | 31 |
|  |  | 15 | 43 |  | 103 | 95 |  | 21 | 14 |  | 21 | 12 |  | 28 | 32 |
|  | Mean | 13 | 30.7 |  | 110.3 | 87.3 |  | 16.7 | 14.0 |  | 21.0 | 10.7 |  | 28.0 | 29.3 |
|  | SD | 2.6 | 10.7 |  | 11.0 | 7.5 |  | 4.5 | 0.0 |  | 1.0 | 2.3 |  | 6.0 | 3.8 |
|  | MF | 0.9 | 0.9 |  | 1.1 | 0.9 |  | 0.8 | 1.0 |  | 1.0 | 0.7 |  | 0.9 | 1.0 |
|  |  |  |  |  |  |  |  |  |  |  |  |  |  |  |  |
| Matrine |  |  |  |  |  |  |  |  |  |  |  |  |  |  |  |
| 625 µg/plate |  | 15 | 26 |  | 119 | 76 |  | 15 | 26 |  | 11 | 22 |  | 33 | 32 |
|  |  | 20 | 25 |  | 84 | 90 |  | 18 | 16 |  | 17 | 20 |  | 29 | 29 |
|  |  | 11 | 28 |  | 91 | 93 |  | 18 | 10 |  | 16 | 20 |  | 29 | 26 |
|  | Mean | 15.3 | 26.3 |  | 98.0 | 86.3 |  | 17.0 | 17.3 |  | 14.7 | 20.7 |  | 30.3 | 29.0 |
|  | SD | 4.5 | 1.5 |  | 18.5 | 9.1 |  | 1.7 | 8.1 |  | 3.2 | 1.2 |  | 2.3 | 3.0 |
|  | MF | 1.1 | 0.8 |  | 1.0 | 0.9 |  | 0.9 | 1.2 |  | 0.7 | 1.4 |  | 1.0 | 1.0 |
|  |  |  |  |  |  |  |  |  |  |  |  |  |  |  |  |
| Matrine |  |  |  |  |  |  |  |  |  |  |  |  |  |  |  |
| 312.5 µg/plate |  | 24 | 26 |  | 104 | 98 |  | 23 | 15 |  | 15 | 20 |  | 22 | 31 |
|  |  | 20 | 32 |  | 94 | 104 |  | 17 | 15 |  | 13 | 14 |  | 33 | 18 |
|  |  | 12 | 32 |  | 96 | 82 |  | 23 | 14 |  | 13 | 23 |  | 27 | 29 |
|  | Mean | 18.7 | 30.0 |  | 98.0 | 94.7 |  | 21.0 | 14.7 |  | 13.7 | 19.0 |  | 27.3 | 26.0 |
|  | SD | 6.1 | 3.5 |  | 5.3 | 11.4 |  | 3.5 | 0.6 |  | 1.2 | 4.6 |  | 5.5 | 7.0 |
|  | MF | 1.3 | 0.9 |  | 1.0 | 1.0 |  | 1.1 | 1.0 |  | 0.7 | 1.3 |  | 0.9 | 0.9 |
|  |  |  |  |  |  |  |  |  |  |  |  |  |  |  |  |
| Solvent control |  |  |  |  |  |  |  |  |  |  |  |  |  |  |  |
|  |  | 13 | 35 |  | 89 | 91 |  | 23 | 11 |  | 28 | 15 |  | 40 | 27 |
|  |  | 11 | 31 |  | 112 | 104 |  | 18 | 17 |  | 18 | 15 |  | 19 | 33 |
|  |  | 19 | 31 |  | 97 | 91 |  | 19 | 14 |  | 16 | 14 |  | 33 | 24 |
|  | Mean | 14.3 | 32.3 |  | 99.3 | 95.3 |  | 20.0 | 14.0 |  | 20.7 | 14.7 |  | 30.7 | 28.0 |
|  | SD | 4.2 | 2.3 |  | 11.7 | 7.5 |  | 2.6 | 3.0 |  | 6.4 | 0.6 |  | 10.7 | 4.6 |
|  | MF | 1.0 | 1.0 |  | 1.0 | 1.0 |  | 1.0 | 1.0 |  | 1.0 | 1.0 |  | 1.0 | 1.0 |
|  |  |  |  |  |  |  |  |  |  |  |  |  |  |  |  |
| Positive control |  |  |  |  |  |  |  |  |  |  |  |  |  |  |  |
|  |  | 342 | 402 |  | 590 | 1159 |  | 681 | 42 |  | 482 | 44 |  | 371 | 65 |
|  |  | 397 | 334 |  | 653 | 1285 |  | 882 | 44 |  | 484 | 47 |  | 347 | 64 |
|  |  | 468 | 356 |  | 670 | 1272 |  | 864 | 46 |  | 758 | 50 |  | 367 | - |
|  | Mean | 402.3 | 364.0 |  | 637.7 | 1238.7 |  | 809.0 | 44.0 |  | 574.7 | 47.0 |  | 361.7 | 64.5 |
|  | SD | 63.2 | 34.7 |  | 42.1 | 69.3 |  | 111.2 | 2.0 |  | 158.8 | 3.0 |  | 12.9 | 0.7 |
|  | MF | 28.1 | 11.3 |  | 6.4 | 13.0 |  | 40.5 | 3.1 |  | 27.8 | 3.2 |  | 11.8 | 2.3 |
|  |  | 2-NF | 2-AA |  | NaN_3_ | 2-AA |  | NaN_3_ | 2-AA |  | 9-AA | 2-AA |  | 4-NQO | 2-AA |
|  | µg/plate | 4 | 2 |  | 2.5 | 5 |  | 2.5 | 2 |  | 25 | 2 |  | 1 | 10 |

| **Table VI.** Revertant counts in the pre-incubation experiment for oxymatrine | | | | | | | | | | | | | | | |
| --- | --- | --- | --- | --- | --- | --- | --- | --- | --- | --- | --- | --- | --- | --- | --- |
|  |  | TA98 |  |  | TA100 |  |  | TA1535 | |  | TA1537 | |  | E.coli WP2 uvrA | |
|  |  | -S9 | +S9 |  | -S9 | +S9 |  | -S9 | +S9 |  | -S9 | +S9 |  | -S9 | +S9 |
| Oxymatrine |  |  |  |  |  |  |  |  |  |  |  |  |  |  |  |
| 5000 µg/plate |  | 46 | 23 |  | 103 | 116 |  | 17 | 13 |  | 17 | 13 |  | 28 | 40 |
|  |  | 40 | 27 |  | 104 | 98 |  | 18 | 9 |  | 18 | 9 |  | 29 | 29 |
|  |  | 26 | 23 |  | 92 | 104 |  | 19 | 13 |  | 19 | 13 |  | 21 | 20 |
|  | Mean | 37.3 | 24.3 |  | 99.7 | 106 |  | 18 | 11.7 |  | 18.0 | 11.7 |  | 26.0 | 29.7 |
|  | SD | 10.3 | 2.3 |  | 6.7 | 9.2 |  | 1 | 2.3 |  | 1.0 | 2.3 |  | 4.4 | 10 |
|  | MF | 1.3 | 1 |  | 1.1 | 1.2 |  | 0.8 | 0.9 |  | 0.8 | 0.9 |  | 1.0 | 0.9 |
|  |  |  |  |  |  |  |  |  |  |  |  |  |  |  |  |
| Oxymatrine |  |  |  |  |  |  |  |  |  |  |  |  |  |  |  |
| 3750 µg/plate |  | 29 | 28 |  | 92 | 98 |  | 14 | 18 |  | 14 | 18 |  | 20 | 27 |
|  |  | 28 | 22 |  | 118 | 85 |  | 17 | 12 |  | 17 | 12 |  | 25 | 36 |
|  |  | 37 | 32 |  | 114 | 79 |  | 16 | 21 |  | 16 | 21 |  | 20 | 12 |
|  | Mean | 31.3 | 27.3 |  | 108 | 87.3 |  | 15.7 | 17 |  | 15.7 | 17 |  | 21.7 | 25 |
|  | SD | 4.9 | 5.0 |  | 14.0 | 9.7 |  | 1.5 | 4.6 |  | 1.5 | 4.6 |  | 2.9 | 12.1 |
|  | MF | 1.1 | 1.1 |  | 1.2 | 1.0 |  | 0.7 | 1.2 |  | 0.7 | 1.2 |  | 0.8 | 0.8 |
|  |  |  |  |  |  |  |  |  |  |  |  |  |  |  |  |
| Oxymatrine |  |  |  |  |  |  |  |  |  |  |  |  |  |  |  |
| 2500 µg/plate |  | 34 | 33 |  | 111 | 99 |  | 16 | 17 |  | 16 | 17 |  | 25 | 34 |
|  |  | 28 | 34 |  | 105 | 104 |  | 25 | 15 |  | 25 | 15 |  | 31 | 25 |
|  |  | 32 | 24 |  | 81 | 113 |  | 24 | 16 |  | 24 | 16 |  | 16 | 26 |
|  | Mean | 31.3 | 30.3 |  | 99.0 | 105.3 |  | 21.7 | 16.0 |  | 21.7 | 16.0 |  | 24.0 | 28.3 |
|  | SD | 3.1 | 5.5 |  | 15.9 | 7.1 |  | 4.9 | 1.0 |  | 4.9 | 1.0 |  | 7.5 | 4.9 |
|  | MF | 1.1 | 1.2 |  | 1.1 | 1.2 |  | 0.9 | 1.2 |  | 0.9 | 1.2 |  | 0.9 | 0.9 |
|  |  |  |  |  |  |  |  |  |  |  |  |  |  |  |  |
| Oxymatrine |  |  |  |  |  |  |  |  |  |  |  |  |  |  |  |
| 1250 µg/plate |  | 33 | 35 |  | 89 | 109 |  | 19 | 16 |  | 19 | 16 |  | 24 | 22 |
|  |  | 43 | 29 |  | 78 | 106 |  | 20 | 19 |  | 20 | 19 |  | 19 | 22 |
|  |  | 28 | 23 |  | 83 | 98 |  | 17 | 17 |  | 17 | 17 |  | 28 | 27 |
|  | Mean | 34.7 | 29 |  | 83.3 | 104.3 |  | 18.7 | 17.3 |  | 18.7 | 17.3 |  | 23.7 | 23.7 |
|  | SD | 7.6 | 6.0 |  | 5.5 | 5.7 |  | 1.5 | 1.5 |  | 1.5 | 1.5 |  | 4.5 | 2.9 |
|  | MF | 1.2 | 1.2 |  | 0.9 | 1.2 |  | 0.8 | 1.3 |  | 0.8 | 1.3 |  | 0.9 | 0.7 |
|  |  |  |  |  |  |  |  |  |  |  |  |  |  |  |  |
| Oxymatrine |  |  |  |  |  |  |  |  |  |  |  |  |  |  |  |
| 625 µg/plate |  | 30 | 23 |  | 98 | 100 |  | 14 | 12 |  | 14 | 12 |  | 24 | 34 |
|  |  | 30 | 31 |  | 113 | 110 |  | 14 | 11 |  | 14 | 11 |  | 35 | 29 |
|  |  | 27 | 29 |  | 103 | 98 |  | 25 | 8 |  | 25 | 8 |  | 23 | 33 |
|  | Mean | 29 | 27.7 |  | 104.7 | 102.7 |  | 17.7 | 10.3 |  | 17.7 | 10.3 |  | 27.3 | 32.0 |
|  | SD | 1.7 | 4.2 |  | 7.6 | 6.4 |  | 6.4 | 2.1 |  | 6.4 | 2.1 |  | 6.7 | 2.6 |
|  | MF | 1.0 | 1.1 |  | 1.2 | 1.2 |  | 0.8 | 0.8 |  | 0.8 | 0.8 |  | 1.0 | 1.0 |
|  |  |  |  |  |  |  |  |  |  |  |  |  |  |  |  |
| Oxymatrine |  |  |  |  |  |  |  |  |  |  |  |  |  |  |  |
| 312.5 µg/plate |  | 42 | 28 |  | 111 | 77 |  | 18 | 12 |  | 18 | 12 |  | 27 | 25 |
|  |  | 30 | 20 |  | 100 | 97 |  | 19 | 14 |  | 19 | 14 |  | 31 | 26 |
|  |  | 24 | 28 |  | 89 | 98 |  | 19 | 15 |  | 19 | 15 |  | 18 | 28 |
|  | Mean | 32 | 25.3 |  | 100.0 | 90.7 |  | 18.7 | 13.7 |  | 18.7 | 13.7 |  | 25.3 | 26.3 |
|  | SD | 9.2 | 4.6 |  | 11.0 | 11.8 |  | 0.6 | 1.5 |  | 0.6 | 1.5 |  | 6.7 | 1.5 |
|  | MF | 1.1 | 1.0 |  | 1.1 | 1.0 |  | 0.8 | 1.0 |  | 0.8 | 1.0 |  | 0.9 | 0.8 |
|  |  |  |  |  |  |  |  |  |  |  |  |  |  |  |  |
| Solvent control |  |  |  |  |  |  |  |  |  |  |  |  |  |  |  |
|  |  | 31 | 27 |  | 91 | 103 |  | 20 | 10 |  | 20 | 10 |  | 30 | 37 |
|  |  | 30 | 21 |  | 81 | 84 |  | 29 | 11 |  | 29 | 11 |  | 26 | 29 |
|  |  | 25 | 26 |  | 96 | 79 |  | 20 | 20 |  | 20 | 20 |  | 26 | 30 |
|  | Mean | 28.7 | 24.7 |  | 89.3 | 88.7 |  | 23.0 | 13.7 |  | 23.0 | 13.7 |  | 27.3 | 32.0 |
|  | SD | 3.2 | 3.2 |  | 7.6 | 12.7 |  | 5.2 | 5.5 |  | 5.2 | 5.5 |  | 2.3 | 4.4 |
|  | MF | 1.0 | 1.0 |  | 1.0 | 1.0 |  | 1.0 | 1.0 |  | 1.0 | 1.0 |  | 1.0 | 1.0 |
|  |  |  |  |  |  |  |  |  |  |  |  |  |  |  |  |
| Positive control |  |  |  |  |  |  |  |  |  |  |  |  |  |  |  |
|  |  | 493 | 387 |  | 663 | 1019 |  | 572 | 62 |  | 572 | 62 |  | 302 | 65 |
|  |  | 478 | 346 |  | 426 | 1277 |  | 567 | 40 |  | 567 | 40 |  | 344 | 88 |
|  |  | 553 | 347 |  | 792 | 1164 |  | 611 | 43 |  | 611 | 43 |  | 342 | - |
|  | Mean | 508 | 360 |  | 627 | 1153.3 |  | 583.3 | 48.3 |  | 583.3 | 48.3 |  | 329.3 | 76.5 |
|  | SD | 39.7 | 23.4 |  | 185.6 | 129.3 |  | 24.1 | 11.9 |  | 24.1 | 11.9 |  | 23.7 | 45.6 |
|  | MF | 17.7 | 14.6 |  | 7.0 | 13.0 |  | 25.4 | 3.5 |  | 25.4 | 3.5 |  | 12.0 | 2.4 |
|  |  | 2-NF | 2-AA |  | NaN_3_ | 2-AA |  | NaN_3_ | 2-AA |  | 9-AA | 2-AA |  | 4-NQO | 2-AA |
|  | µg/plate | 4 | 2 |  | 2.5 | 5 |  | 2.5 | 2 |  | 25 | 2 |  | 1 | 10 |
